# Editorial: Defining the Spatial Organization of Immune Responses to Cancer and Viruses *In Situ*

**DOI:** 10.3389/fimmu.2022.847582

**Published:** 2022-01-24

**Authors:** Darci Phillips, Scott J. Rodig, Sizun Jiang

**Affiliations:** ^1^ Department of Pathology, Stanford University School of Medicine, Stanford, CA, United States; ^2^ Department of Dermatology, Stanford University School of Medicine, Stanford, CA, United States; ^3^ Department of Pathology, Brigham and Women’s Hospital, Boston, MA, United States; ^4^ Department of Oncologic Pathology, Dana Farber Cancer Institute, Boston, MA, United States; ^5^ Center for Virology and Vaccine Research, Beth Israel Deaconess Medical Center, Boston, MA, United States

**Keywords:** spatial organization, cancer biology, viruses, immune checkpoint inhibitors, biothreat agents, tissue imaging, cell segmentation and classification

Cellular organization within tissues is purposeful: specific cell-types are arranged at different proximities with intent, thus enabling intercellular crosstalk and driving tissue functions in health, disease, and response to therapy. Elucidating the spatial pattern of cells and molecules within their native tissue microenvironment is therefore critical towards identifying tissue-based biomarkers predictive of clinical outcome.

Multiplexed tissue imaging methods—including imaging mass cytometry (IMC) (1), multiplex ion beam imaging (MIBI) (2, 3), multiplex immunohistochemistry (mIHC) (4), CO-Detection by indEXing (CODEX) (5, 6), cyclic immunofluorescence (CyCIF) (7, 8), and spatial transcriptomics (9–12)—allow for the simultaneous detection of more than 50 proteins and 1000s of transcripts, thereby empowering the interrogation of spatial organization within tissues. Importantly, these technologies retain the native tissue context of each individual cell, while enabling deep phenotypical and functional interrogation. To date, these methods have enhanced our understanding of the diverse tissue microenvironments in oncology (3, 13–19), reactive and auto-immunology (5, 20, 21), and microbiology (22–24).

This Research Topic focuses on the spatial organization of immune responses to cancer and viral infections. It brings together nine manuscripts that 1) contribute methods to improve the accuracy of cell-type annotation, 2) provide new computational tools to profile spatial tissue patterns, and 3) advance our understanding of spatially resolved immune responses to cancer, infections, and immunotherapy.

A well-designed multiplexed antibody panel is critical for accurate cell-type annotation and serves as the foundation for characterizing cellular composition, cell-cell interactions, intracellular functional states and all further downstream spatial analyses (Phillips D. et al.) optimize a 56-marker CODEX panel consisting of major structural, tumor, and immune cell markers, including eight regulatory proteins that are common immunotherapy targets—PD-1, PD-L1, CTLA-4, ICOS, IDO-1, LAG-3, OX40, TIM-3, and VISTA. As such, this panel provides an important tool for informing clinical cancer care and the design of therapeutic combination strategies across tumor types. Jiang S. et al. present a 21-marker CODEX panel consisting of 18 antibodies for major immune cell-types and 3 Ebola virus-specific antibodies in rhesus macaques. Importantly, this is one of the first highly multiplexed tissue imaging antibody panels targeted towards rhesus macaques, a common non-human primate model used to evaluate the efficacy of medical countermeasures against biothreat pathogens. Development and optimization of both multiplexed antibody panels was costly and time-consuming, but these panels are easy to reproduce, expand upon, and translate towards other multiplexed imaging modalities. For example, the analogous biochemistry of CODEX and MIBI/IMC antibody conjugations have allowed many of our stains to be reproduced across these platforms (unpublished observations from D.P. and S.J.). Thus, these studies provide investigators with a solid starting point for interrogating how cells functionally organize within tissues to mount coordinated immune responses to cancer, infections, biothreats, and therapeutic intervention.

Accurate cell-type identification is also heavily influenced by chosen normalization strategy and data pre-processing algorithms. Hickey et al. evaluate the performance of major normalization techniques (i.e., Z, log(double Z), min-max, and arcsinh) in mitigating the effects of noise on cell-type annotation in a CODEX dataset. This study shows that regardless of the downstream unsupervised clustering algorithm used, Z normalization of marker intensity results in the most reproducible intra- and inter-sample comparisons for the most accurate cell-type annotation. Correct cell-type identification also depends on the ability to minimize and correct for lateral signal spillover from adjacent cells, a particular challenge in packed lymphoid or tumor tissues. Given the large number of antibody tags imaged in multiplexed experiments, cumulative pairwise spillovers in densely packed tissues can have detrimental effects on cell-type assignments and downstream biological conclusions. Bai et al. present a lateral spillover compensation algorithm termed Reinforcement Dynamic Spillover EliminAtion (REDSEA), which allows robust reassignment of lateral spillover signal to the cell of origin based on the proportion of the shared boundary between adjacent cells. Application of REDSEA to MIBI and CyCIF datasets led to significant improvement in cell-type annotation (i.e., 56.0% of cells were correctly identified at baseline compared to 81.5% after a border-based REDSEA compensation). These studies provide platform agnostic image processing tools that increase the certainty of marker intensities extracted from individual segmented cells, thereby improving the speed and accuracy of cell-type identification.

In addition to REDSEA (Bai et al.), this Research Topic provides additional computational tools that resolve cellular tissue heterogeneity and reveal complex tissue architecture. Yuan et al. present Seg-SOM, a computer vision method for dimensionality reduction of nuclear morphology in histological images. Seg-SOM is easily scalable: it is entirely automated, performs dimensionality reduction on hundreds of thousands of cells within seconds, and can operate on H&E-stained or multiplexed images. Application of Seg-SOM to breast cancer imaging datasets enabled the 1) prediction of tumor-infiltrating lymphocyte density in normal and cancerous breast tissues and 2) classification of ductal carcinoma *in situ* lesions into those that exist in isolation or were accompanied by invasive breast cancer. Baranwal et al. present Cell-Graph Attention (CGAT) Network, a graph-theory approach that allows for grading of pancreatic disease based on point patterns derived from multiplexed immunofluorescence-stained images. The CGAT framework can differentiate pancreatic ductal adenocarcinoma from chronic pancreatitis. This is of fundamental clinical impact as the similar pathological appearances of these conditions can often lead to either a missed diagnosis of an aggressive cancer or repeated, unnecessary biopsies of a benign condition. Modular implementation of these computational approaches into existing analytical pipelines will provide new avenues of investigation and facilitate a greater understanding of spatial dynamics in complex tissue microenvironments.

A major effort of multiplexed tissue imaging is to better understand how the composition and spatial interactions of distinct cell-types in tumor tissues contribute to disease prognosis and response predictions during immunotherapy. Stoltzfus et al. leveraged upon multi-parameter confocal imaging, histocytometry, and a previous described computational method for distinguishing tissue organization, CytoMAP (30), to characterize immune cell organization in mouse models of colorectal and pancreatic cancer to identify a perivascular immune niche (i.e., co-localization of myeloid and CD8^+^ T cell aggregates adjacent to tumor blood vessels), which is positively associated with anti-PD-L1 immunotherapy response. The increased abundance of this perivascular immune niche following immunotherapy suggests a major role of blood vessels in coordinating the active remodeling of innate and adaptive immune cells within tumors, leading to improved antitumor immunity. Ardighieri et al. combine immunohistochemistry with RNAscope to identify a subset of M1-type tumor-associated macrophages (TAMs) (i.e., CXCL10^+^IRF1^+^STAT1^+^) in ovarian cancer. Patients with a high density of these tumor-infiltrating macrophages have improved prognoses and superior responses to platinum-based therapies. These findings are also extended to other cancer types—including melanoma, head and neck squamous cell carcinoma (HNSCC), colorectal cancer, endometrial cancer, breast cancer, and lung cancer—suggesting that these specialized M1-polarized TAMs are part of a T-cell infiltrated immune contexture that confers a better clinical outcome. Yoshimura et al. utilized multiplexed immunohistochemistry and image cytometry-based quantification to reveal co-localization of PD-1^+^ helper T cells and CD163^+^ TAMs within *tumor cells nests* as a negative prognostic indicator in HNSCC. This finding suggests that CD163^+^ TAMs exert their immunosuppressive effects on effector PD-1^+^ helper T cells, in line with recent orthogonal work showing that co-localization of PD-1^+^CD4^+^ T cells and Tregs was associated with poor response to immunotherapy (18). Collectively, these studies provide a framework for utilizing advanced spatial analyses to interrogate the complexity of the tumor microenvironment and decode the therapeutic response *in situ*.

For multiplexed tissue imaging to reach its full potential as a research paradigm, it is pertinent that these studies are not performed in isolation. Orthogonal interrogation with synergistic tools, such as RNA quantification methods including spatial transcriptomics, single-cell RNA sequencing, and other advanced techniques are needed to precisely define the genetic and protein topographies of human tissues. The development of multi-omic measurements *in situ* is a key fundamental advancement in this regard, including the quantification of metabolic states (21), nucleic acids and proteins (12, 23, 25), clonality (26), and epigenetic states (27). The continued advancement of computational methods is also vital for multi-scalar inferential analysis across different measurement modalities (28). Future studies must be performed in large, well-annotated clinical cohorts to delineate more subtle features of the tissue’s spatial architecture and to determine if spatial findings translate broadly, with the incorporation of powerful statistical frameworks to aid in experimental designs (29). To this end, numerous challenges must be overcome, including 1) establishing protocols for collecting tissue specimens that minimize fixation artifacts, 2) compilation of published lists of domain expert-verified antibody clones that retain specificity even after conjugation, 3) development of better methods for segmentation, normalization and quantification of single-cell protein expression intensities, 4) novel algorithms to automate cell-type and tissue feature identification in a scalable manner, 5) experimental and computational methods to enable multi-modal measurements of spatial-resolved single cells.

In sum, investigation into the spatial organization of cells and molecules within tissues is advancing at a rapid and exciting pace. The articles in this Research Topic serve as a reference for those interested in using multiplexed tissue imaging technologies and emerging computational tools to enable a comprehensive understanding of tissue-level immune responses to cancer, viral infections, and immunotherapy.

## Author Contributions

All authors wrote and revised the manuscript. All authors approved the submitted version.

## Funding

This work was supported by the National Institutes of Health (NIH) F32CA233203 (DP) and R01AI149672 (SJ), The Beckman Center for Molecular and Genetic Medicine (DP), Bristol-Myers Squibb (SJR), Merck (SJR), KITE/Gilead (SJR), and the Bill & Melinda Gates Foundation INV-002704 (SJ). SJR is also a Scientific Advisory Board member for KITE/Gilead and Immunitas Therapeutics.

## Conflict of Interest

The authors declare that the research was conducted in the absence of any commercial or financial relationships that could be construed as a potential conflict of interest.

## Publisher’s Note

All claims expressed in this article are solely those of the authors and do not necessarily represent those of their affiliated organizations, or those of the publisher, the editors and the reviewers. Any product that may be evaluated in this article, or claim that may be made by its manufacturer, is not guaranteed or endorsed by the publisher.
